# Supplementary material for: Food inflation and child undernutrition in low and middle income countries
Source: Nat Commun. 2023 Sep 16;14:5761. doi: 10.1038/s41467-023-41543-9 (PMC10505228; doi:10.1038/s41467-023-41543-9)
Supplement: Supplementary file 1 — Supplementary Information File [file 41467_2023_41543_MOESM1_ESM.pdf]

Supplementary Information

Food inflation and child undernutrition in low and middle income countries

Derek Headey[1*], Marie Ruel[2]

1. Senior Research Fellow, The International Food Policy Research Institute (IFPRI), Colombo Sri Lanka

2. Senior Research Fellow, The International Food Policy Research Institute (IFPRI), Washington DC USA

*Correspondence: d.headey@cgiar.org

Contents

Supplementary Tables 3

Supplementary Table 1. Descriptive statistics for key variables 3

Supplementary Table 2. Summary statistics for additional country level control variables used in robustness tests 4

Supplementary Table 3. Mean prevalence of wasting and stunting prevalence by DHS round 5

Supplementary Table 4. Sample sizes and population shares pooled by country 8

Supplementary Table 5. Weighted multivariate linear probability models of severe wasting risks as a function of 5% increases in the real food price index over the past 3 months interacted with urban locality, gender, asset poverty and farmland ownership 9

Supplementary Table 6. Weighted multivariate linear probability models of wasting risks as a function of 5% increases in the real food price index over the past 3 months, controlling for other national level indicators 10

Supplementary Table 7. Weighted multivariate linear probability models of stunting risks as a function of 5% increases in the real food price index in the first 1000 days of life, interacted with urban locality, gender, asset poverty and farmland ownership 11

Supplementary Figures 13

Supplementary Figure 1. Local polynomial estimates of wasting (WHZ< -2) prevalence against child age in months for various DHS strata 13

Supplementary Figure 2. Local polynomial estimates of wasting (WHZ< -2) prevalence against child age in months for children from asset-poor and non-poor households in India 15

Supplementary Figure 3. A histogram of real food price changes in the 3 months prior to wasting measurement 16

Supplementary Figure 4. Weighted multivariate linear probability models of wasting and stunting risks as a function of poor diet diversity in the past 24 hours and diarrhea or fever in the past two weeks 17

Supplementary Figure 5. Weighted multivariate linear probability coefficients of wasting and severe wasting as a function of both food inflation and total inflation over the past 3 months 18

Supplementary Figure 6. Weighted multivariate linear probability coefficients of stunting and severe stunting for children 24-59 months as a function of food inflation and total inflation at various stages of the first 1000 days of life 19

Supplementary Tables

Supplementary Table 1. Descriptive statistics for key variables

Variable	N	Mean	Std. Dev.	Min	Max
Nutrition outcomes					
Wasting (WHZ<-2)	1,271,886	0.13	0.33	0	1
Severe Wasting (WHZ<-3)	1,271,886	0.05	0.22	0	1
Stunting (HAZ <-2)	1,254,633	0.35	0.48	0	1
Severe Stunting (HAZ <-3)	1,254,633	0.15	0.35	0	1
National-level indicators					
Real food inflation, last 3 months	1,271,886	-0.10	2.24	-10.67	13.71
Real food inflation, last 12 months	1,271,886	0.25	3.10	-10.32	19.96
Individual, mother, household indicators					
Household owns no DHS assets (asset-poor)	1,271,886	0.26	0.44	0	1
Household owns some (< 5 y) assets	1,271,886	0.63	0.48	0	1
Household owns all 5 assets	1,271,886	0.11	0.31	0	1
Mother has 9 or more years of schooling	1,271,886	0.32	0.47	0	1
Household has piped water	1,271,886	0.39	0.49	0	1
Household has flush toilet	1,271,886	0.35	0.48	0	1
Child born in medical facility	1,271,886	0.66	0.47	0	1
Mother received 4 or more ANC visits	1,271,886	0.70	0.46	0	1
Child received all vaccinations	1,271,886	0.42	0.49	0	1
Teenage mother (at birth)	1,271,886	0.17	0.38	0	1
Mother has 4 or more children	1,271,886	0.34	0.47	0	1
Household is rural	1,271,886	0.70	0.46	0	1
Child is girl	1,271,886	0.49	0.50	0	1
Household owns farmland	1,049,200	0.54	0.50	0	1
Poor diet in past 24 hours (<4 of 7 foods)	327,522	0.71	0.55	0	1
Diarrhea in past 2 weeks	1,265,488	0.13	0.33	0	1
Fever in past 2 weeks	1,257,720	0.20	0.40	0	1

Source: Authors' estimates. See text for details on sources.

Supplementary Table 2. Summary statistics for additional country level control variables used in robustness tests

Variable	N	Mean	Std. dev.	Min	Max
Growth in broad money (%)	125	15.95	10.70	-9.30	69.08
Battle deaths per 100,000 people	129	107.55	324.23	0.00	1817.00
Growth in GDP per capita (%)	130	0.00	0.00	-0.01	0.02
Growth in food production (%)	130	0.04	0.07	-0.23	0.31
Growth in terms of trade (%)	130	0.03	0.09	-0.39	0.32

Source: Authors' estimates. See text for details on sources.

Supplementary Table 3. Mean prevalence of wasting and stunting prevalence by DHS round

Country	Survey start	Survey end	Mod/severe wasting	Mod/severe stunting
Albania	2008	2009	10.0%	23.9%
Albania	2017	2018	2.2%	12.6%
Armenia	2005	2005	5.3%	15.5%
Armenia	2010	2010	4.2%	22.2%
Armenia	2015	2016	5.3%	10.4%
Bangladesh	2004	2004	14.6%	49.6%
Bangladesh	2007	2007	17.2%	41.6%
Bangladesh	2011	2011	15.9%	40.6%
Bangladesh	2014	2014	14.7%	36.6%
Bangladesh	2017	2018	8.8%	31.3%
Benin	2001	2001	9.9%	38.6%
Benin	2006	2006	9.0%	43.4%
Benin	2011	2012	17.5%	47.0%
Benin	2017	2018	5.3%	32.9%
Bolivia	2003	2003	2.2%	33.5%
Bolivia	2008	2008	1.7%	26.4%
Burkina Faso	2003	2003	24.1%	42.9%
Burkina Faso	2010	2010	16.0%	34.2%
Burundi	2010	2010	6.2%	55.2%
Burundi	2016	2017	5.1%	54.5%
Cambodia	2005	2005	8.6%	44.8%
Cambodia	2010	2010	12.0%	40.1%
Cambodia	2014	2014	10.3%	32.6%
Cameroon	2004	2004	6.2%	35.2%
Cameroon	2011	2011	5.9%	31.7%
Cameroon	2018	2019	4.1%	28.3%
Chad	2004	2004	18.4%	42.7%
Chad	2014	2015	15.1%	42.9%
Colombia	2005	2005	2.1%	15.7%
Colombia	2010	2010	1.1%	14.5%
Congo, Rep.	2005	2005	8.4%	28.8%
Congo, Rep.	2011	2012	5.7%	26.8%
Congo (DRC)	2007	2007	11.0%	45.0%
Congo (DRC)	2013	2014	8.7%	44.0%
Dominican Rep.	2002	2002	2.3%	12.3%
Dominican Rep.	2007	2007	2.2%	11.4%
Dominican Rep.	2013	2013	2.6%	7.6%
Egypt	2003	2003	5.1%	20.1%
Egypt	2005	2005	5.7%	27.2%
Egypt	2008	2008	8.3%	29.7%
Egypt	2014	2014	13.0%	20.1%
Ethiopia	2008	2016	12.4%	36.4%

Ethiopia	2011	2011	12.1%	42.3%
Ghana	2003	2003	9.6%	36.1%
Ghana	2008	2008	10.0%	27.9%
Ghana	2014	2014	5.1%	19.2%
Guinea	2005	2005	11.4%	38.8%
Guinea	2012	2012	11.3%	30.7%
Guinea	2018	2018	9.4%	30.6%
Haiti	2005	2006	9.7%	29.5%
Haiti	2012	2012	5.3%	22.3%
Haiti	2016	2017	3.8%	21.4%
Honduras	2005	2006	1.5%	35.1%
Honduras	2011	2012	1.5%	25.4%
India	2005	2006	19.2%	43.5%
India	2015	2016	21.1%	38.1%
India	2019	2021	18.6%	35.9%
Jordan	2002	2002	2.5%	13.2%
Jordan	2007	2007	9.1%	16.1%
Jordan	2009	2009	1.7%	10.2%
Jordan	2012	2012	2.4%	8.8%
Kenya	2003	2003	7.6%	34.5%
Kenya	2008	2009	8.9%	34.2%
Kenya	2014	2014	5.8%	27.1%
Lesotho	2004	2004	6.1%	44.2%
Lesotho	2009	2010	4.8%	39.4%
Lesotho	2014	2014	3.8%	34.5%
Liberia	2007	2007	8.3%	38.3%
Liberia	2013	2013	6.9%	31.1%
Malawi	2004	2004	6.9%	52.3%
Malawi	2010	2010	4.5%	46.6%
Malawi	2015	2016	3.4%	35.5%
Maldives	2009	2009	12.3%	19.0%
Maldives	2016	2017	9.5%	15.1%
Mali	2001	2001	13.7%	42.1%
Mali	2006	2006	17.2%	38.1%
Mali	2012	2013	14.0%	37.5%
Mali	2018	2018	9.8%	27.5%
Mozambique	2003	2003	5.3%	45.4%
Mozambique	2011	2011	5.7%	39.5%
Namibia	2006	2007	8.5%	29.1%
Namibia	2013	2013	8.8%	22.4%
Nepal	2006	2006	12.8%	50.1%
Nepal	2011	2011	10.9%	41.5%
Nepal	2016	2016	9.8%	36.0%
Niger	2006	2006	13.1%	49.5%

Niger	2012	2012	19.3%	41.9%
Nigeria	2003	2003	12.3%	41.8%
Nigeria	2008	2008	16.5%	42.2%
Nigeria	2013	2013	17.7%	35.9%
Nigeria	2018	2018	6.9%	36.1%
Pakistan	2012	2013	11.0%	45.3%
Pakistan	2017	2018	9.3%	39.3%
Peru	2003	2008	1.0%	29.9%
Peru	2009	2009	0.8%	27.0%
Peru	2010	2010	0.8%	25.5%
Peru	2011	2011	0.6%	23.1%
Peru	2012	2012	0.7%	21.0%
Rwanda	2005	2005	4.9%	49.9%
Rwanda	2010	2010	3.1%	43.8%
Rwanda	2014	2015	2.4%	37.6%
Senegal	2005	2005	9.6%	20.8%
Senegal	2010	2011	10.2%	30.5%
Senegal	2012	2013	10.6%	20.2%
Senegal	2014	2014	7.0%	21.6%
Senegal	2015	2015	8.3%	22.0%
Senegal	2016	2016	7.6%	19.1%
Senegal	2017	2017	9.6%	19.2%
Senegal	2019	2019	9.2%	19.5%
Sierra Leone	2008	2008	12.3%	34.8%
Sierra Leone	2013	2013	10.5%	37.7%
Sierra Leone	2019	2019	5.8%	30.1%
Tajikistan	2012	2012	10.5%	25.2%
Tajikistan	2017	2017	6.7%	18.4%
Tanzania	2004	2005	4.5%	42.7%
Tanzania	2010	2010	6.9%	39.9%
Tanzania	2015	2016	5.1%	33.5%
Timor-Leste	2009	2010	20.8%	56.6%
Timor-Leste	2016	2016	25.7%	46.1%
Uganda	2000	2001	5.7%	44.9%
Uganda	2006	2006	7.0%	38.4%
Uganda	2011	2011	5.8%	32.5%
Uganda	2016	2016	4.0%	28.4%
Zambia	2001	2002	6.4%	53.2%
Zambia	2007	2007	6.6%	44.2%
Zambia	2013	2014	6.9%	39.5%
Zambia	2018	2019	4.4%	34.8%
Zimbabwe	2005	2006	7.6%	34.1%
Zimbabwe	2010	2011	3.7%	31.6%
Zimbabwe	2015	2015	3.8%	25.6%

Source: See Methods and Materials for data sources.

Supplementary Table 4. Sample sizes and population shares pooled by country

Country	DHS observations	Population under-5	Sample share	Population share
Albania	3866	171,796	0.30%	0.06%
Armenia	4205	201,083	0.33%	0.07%
Bangladesh	34000	15,300,000	2.67%	4.95%
Benin	39814	1,558,812	3.13%	0.50%
Bolivia	17005	1,192,606	1.34%	0.39%
Burkina Faso	15218	2,842,946	1.20%	0.92%
Burundi	9534	1,578,653	0.75%	0.51%
Cambodia	11785	1,672,089	0.93%	0.54%
Cameroon	12927	3,462,007	1.02%	1.12%
Chad	14854	2,324,090	1.17%	0.75%
Colombia	28471	3,834,004	2.24%	1.24%
Congo, Rep.	8516	702,728	0.67%	0.23%
Congo, DRC	11719	12,100,000	0.92%	3.92%
Dominican Rep.	21963	989,582	1.73%	0.32%
Egypt	43689	9,744,422	3.43%	3.15%
Ethiopia	18738	14,200,000	1.47%	4.59%
Ghana	8309	3,587,524	0.65%	1.16%
Guinea	9345	1,771,639	0.73%	0.57%
Haiti	12196	1,263,146	0.96%	0.41%
Honduras	18973	1,036,071	1.49%	0.34%
India	477032	128,000,000	37.51%	41.42%
Jordan	20226	1,003,583	1.59%	0.32%
Kenya	28867	6,852,972	2.27%	2.22%
Lesotho	4394	249,721	0.35%	0.08%
Liberia	7679	370,759	0.60%	0.12%
Malawi	18293	2,632,444	1.44%	0.85%
Maldives	4771	33,981	0.38%	0.01%
Mali	34168	2,928,051	2.69%	0.95%
Mozambique	17784	4,176,654	1.40%	1.35%
Namibia	5592	288,161	0.44%	0.09%
Nepal	9987	3,022,827	0.79%	0.98%
Niger	8790	665,690	0.69%	0.22%
Nigeria	62933	27,700,000	4.95%	8.96%
Pakistan	6802	24,000,000	0.53%	7.77%
Peru	46281	3,004,452	3.64%	0.97%
Rwanda	11385	1,578,409	0.90%	0.51%
Senegal	47295	2,147,173	3.72%	0.69%
Sierra Leone	10703	1,054,666	0.84%	0.34%
Tajikistan	10564	987,283	0.83%	0.32%
Tanzania	23117	7,901,238	1.82%	2.56%
Timor-Leste	13843	160,956	1.09%	0.05%
Uganda	11663	6,266,988	0.92%	2.03%
Zambia	31231	2,486,008	2.46%	0.80%
Zimbabwe	13359	1,997,281	1.05%	0.65%

Source: See Methods and Materials for data sources.

Supplementary Table 5. Weighted multivariate linear probability models of severe wasting risks as a function of 5% increases in the real food price index over the past 3 months interacted with urban locality, gender, asset poverty and farmland ownership

	(1)	(2)	(3)	(4)	(5)	(6)
Interaction variable	Poverty	Girl	Urban	Farmland	Multiple	Multiple
Sample	Full	Full	Full	Rural	Urban	Rural
Food inflation	0.22***	0.23***	0.13*	0.42***	0.22***	-0.15***
	(0.06 - 0.38)	(0.08 - 0.39)	(-0.01 - 0.26)	(0.31 - 0.52)	(0.09 - 0.35)	(-0.17 - -0.14)
Food inflation*urban	-0.14***				-0.09**	
	(-0.22 - -0.05)				(-0.16 - -0.02)	
Food inflation*girl		-0.12***			-0.12***	-0.15***
		(-0.15 - -0.08)			(-0.15 - -0.09)	(-0.17 - -0.14)
Food inflation*asset-poor			0.19**		0.17**	0.36***
			(0.03 - 0.36)		(0.01 - 0.32)	(0.26 - 0.47)
Food inflation*farmland				-0.17***		-0.17***
				(-0.24 - -0.11)		(-0.24 - -0.11)
Urban locality				-0.00		-0.00
				(-0.01 - 0.00)		(-0.01 - 0.00)
Girl child	0.03***	0.03***	0.03***	0.03***	0.03***	0.04***
	(0.02 - 0.03)	(0.02 - 0.03)	(0.02 - 0.04)	(0.03 - 0.04)	(0.02 - 0.04)	(0.03 - 0.05)
Asset-poor	0.01***	0.01***	0.01***		0.01***	
	(0.00 - 0.01)	(0.00 - 0.01)	(0.00 - 0.01)		(0.00 - 0.01)	
Farmland ownership	-0.02***	-0.02***	-0.02***	-0.02***	-0.02***	-0.02***
	(-0.02 - -0.01)	(-0.02 - -0.01)	(-0.02 - -0.01)	(-0.02 - -0.01)	(-0.02 - -0.01)	(-0.02 - -0.01)
Observations	1,271,886	1,271,886	1,271,886	719,457	1,271,886	719,457
R-squared	0.06	0.06	0.06	0.05	0.06	0.05

Notes: 95% confidence intervals based on standard errors clustered at the country level are reported in parentheses. Regressions are weighted (See Methods and Materials). Coefficients represent the proportional change in wasting risks from a 5% increase in the real food price index over the past 3 months. The model also incorporates an extensive set of controls described in the Methods and Materials, including DHS variables, and various temporal effects and country fixed effects.

Supplementary Table 6. Weighted multivariate linear probability models of wasting risks as a function of 5% increases in the real food price index over the past 3 months, controlling for other national level indicators

	(1) Base model	(2) Money growth	(3) Conflict (battle deaths)	(5) Exchange rate changes	(6) Food production growth	(7) Terms of trade changes	(8) All country covariates
Food inflation	0.09***	0.09***	0.08***	0.08***	0.08***	0.09***	0.08***
	(0.03 - 0.14)	(0.03 - 0.14)	(0.02 - 0.13)	(0.03 - 0.14)	(0.03 - 0.14)	(0.04 - 0.13)	(0.03 - 0.14)
Broad money		-0.01					-0.02
		(-0.11 - 0.08)					(-0.11 - 0.08)
Battle deaths			1.51				0.33
			(-0.88 - 3.90)				(-1.89 - 2.55)
Exchange rate				-0.03			-0.02
				(-0.11 - 0.04)			(-0.07 - 0.04)
Food production					0.04		0.03
					(-0.07 - 0.15)		(-0.05 - 0.11)
Terms of trade						-0.18**	-0.17**
						(-0.35 - -0.02)	(-0.33 - -0.01)
Observations	1,271,886	1,271,886	1,271,886	1,271,886	1,271,886	1,271,886	1,271,886
R-squared	0.06	0.06	0.06	0.06	0.06	0.06	0.06

Notes: 95% confidence intervals based on standard errors clustered at the country level are reported in parentheses. Regressions are weighted to be representative of the < 5 year population of children of all countries included in this DHS dataset. These coefficients can be interpreted as elasticities, or the proportion change in wasting risks from a 10% increase in the real food price index over the past 3 months. The model also incorporates an extensive set of controls as detailed in the Methods and Materials

Supplementary Table 7. Weighted multivariate linear probability models of stunting risks as a function of 5% increases in the real food price index in the first 1000 days of life, interacted with urban locality, gender, asset poverty and farmland ownership

	(1)	(2)	(3)	(4)
Interactions	Urban	Girl	Asset-poor	Farmland
Sample	Full sample	Full sample	Full Sample	Rural sample
Food inflation, prenatal	0.01*	0.01	0.02***	-0.05*
	(-0.00 - 0.02)	(-0.01 - 0.03)	(0.01 - 0.03)	(-0.11 - 0.01)
Food inflation, 1st year	0.02**	0.02***	0.02**	0.01
	(0.00 - 0.03)	(0.01 - 0.03)	(0.00 - 0.03)	(-0.00 - 0.02)
Food inflation, 2nd year	0.01	0.00	0.02*	0.00
	(-0.01 - 0.03)	(-0.02 - 0.03)	(-0.00 - 0.04)	(-0.01 - 0.02)
Food inflation, prenatal*Urban	0.03***			
	(0.01 - 0.05)			
Food inflation, 1st year*Urban	0.00			
	(-0.01 - 0.02)			
Food inflation, 2nd year*Urban	0.01			
	(-0.03 - 0.05)			
Food inflation, prenatal*Girl		0.01		
		(-0.01 - 0.03)		
Food inflation, 1st year*Girl		0.00		
		(-0.00 - 0.01)		
Food inflation, 2nd year*Girl		0.02*		
		(-0.00 - 0.04)		
Food inflation, prenatal*Asset-poor			-0.01	
			(-0.03 - 0.00)	
Food inflation, 1st year*Asset-poor			0.00	
			(-0.01 - 0.01)	
Food inflation, 2nd year*Asset-poor			-0.01	
			(-0.05 - 0.02)	
Food inflation, prenatal*Farmland				0.00
				(-0.02 - 0.02)
Food inflation, 1st year*Farmland				-0.00
				(-0.01 - 0.01)
Food inflation, 2nd year*Farmland				0.00
				(-0.03 - 0.04)
Urban locality	-0.05	-0.02*	-0.02*	
	(-0.14 - 0.05)	(-0.04 - 0.00)	(-0.04 - 0.00)	

Girl child	-0.01	-0.03**	-0.01	-0.01
	(-0.03 - 0.01)	(-0.05 - -0.00)	(-0.03 - 0.01)	(-0.03 - 0.01)
Asset-poor	0.15***	0.15***	0.15***	0.16***
	(0.13 - 0.18)	(0.13 - 0.18)	(0.07 - 0.24)	(0.15 - 0.18)
Farmland ownership				-0.01
				(-0.11 - 0.09)
Observations	694,673	694,673	694,673	419,768
R-squared	0.10	0.10	0.10	0.07

Notes: 95% confidence intervals based on standard errors clustered at the country level are reported in parentheses. Regressions are weighted (See Methods and Materials). Coefficients represent the proportional change in wasting risks from a 5% increase in the real food price index over the past 3 months. The model also incorporates an extensive set of controls described in the Methods and Materials, including DHS variables, and various temporal effects and country fixed effects.

Supplementary Figures

Supplementary Figure 1. Local polynomial estimates of wasting (WHZ< -2) prevalence against child age in months for various DHS strata

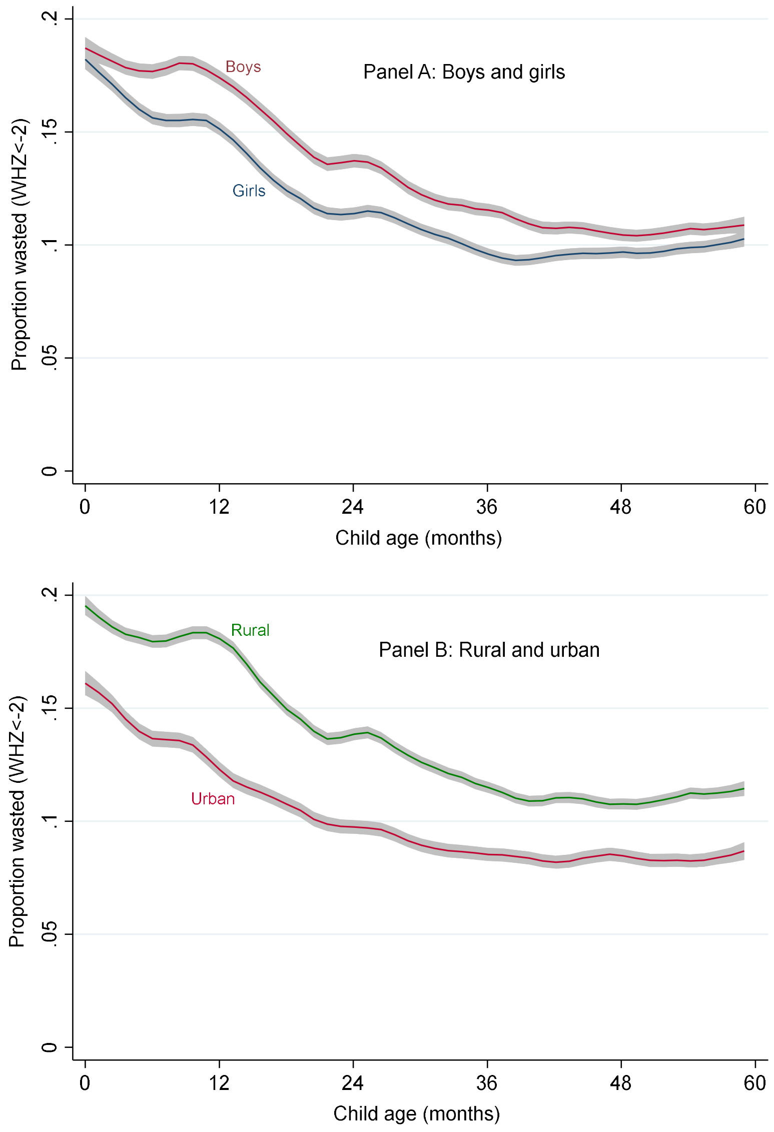

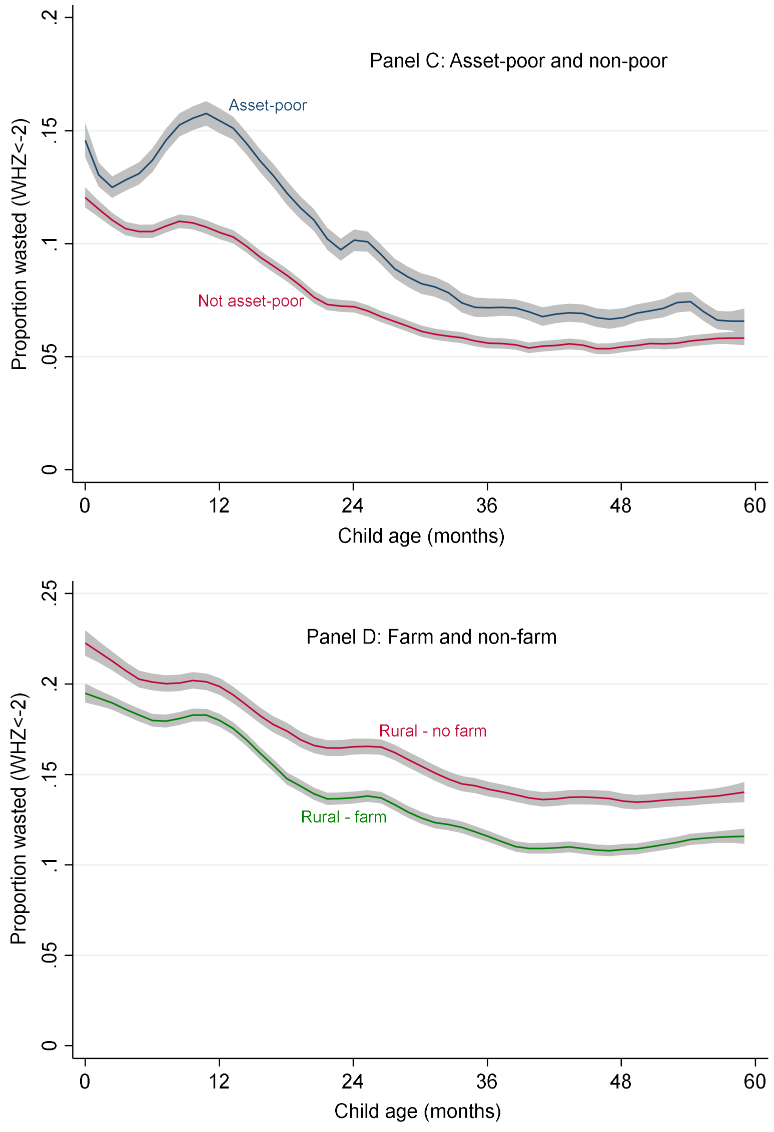

Source: Authors' estimates from DHS data using the *lpoly* command in STATA™. Panel A excludes India, but results for India are reported in Supplement Figure 2.

Supplementary Figure 2. Local polynomial estimates of wasting (WHZ< -2) prevalence against child age in months for children from asset-poor and non-poor households in India

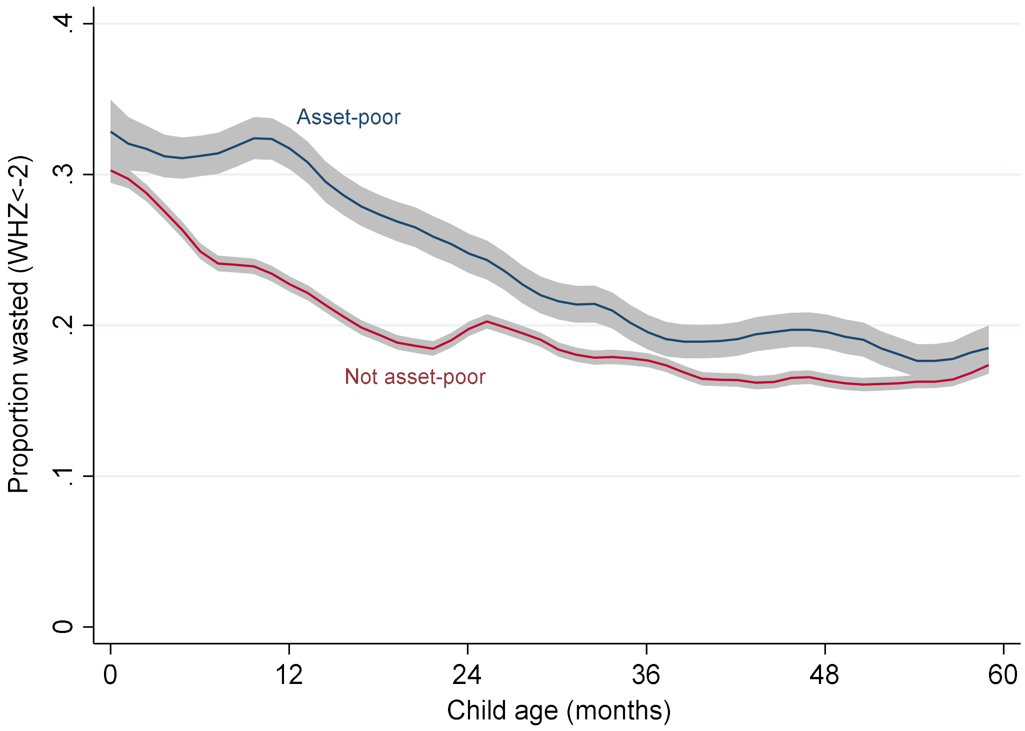

Source: Authors' estimates from DHS data using the lpoly command in STATA™.

Supplementary Figure 3. A histogram of real food price changes in the 3 months prior to wasting measurement

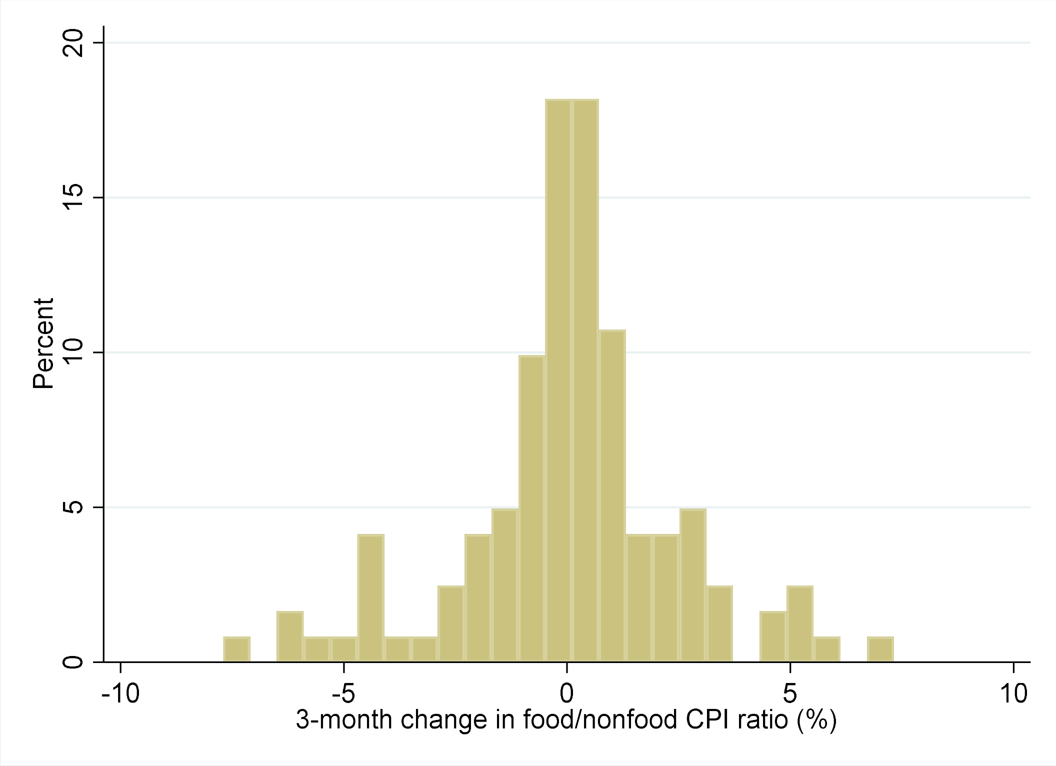

Source: Authors' calculations from FAO consumer price index data.[57]

Supplementary Figure 4. Weighted multivariate linear probability models of wasting and stunting risks as a function of poor diet diversity in the past 24 hours and diarrhea or fever in the past two weeks

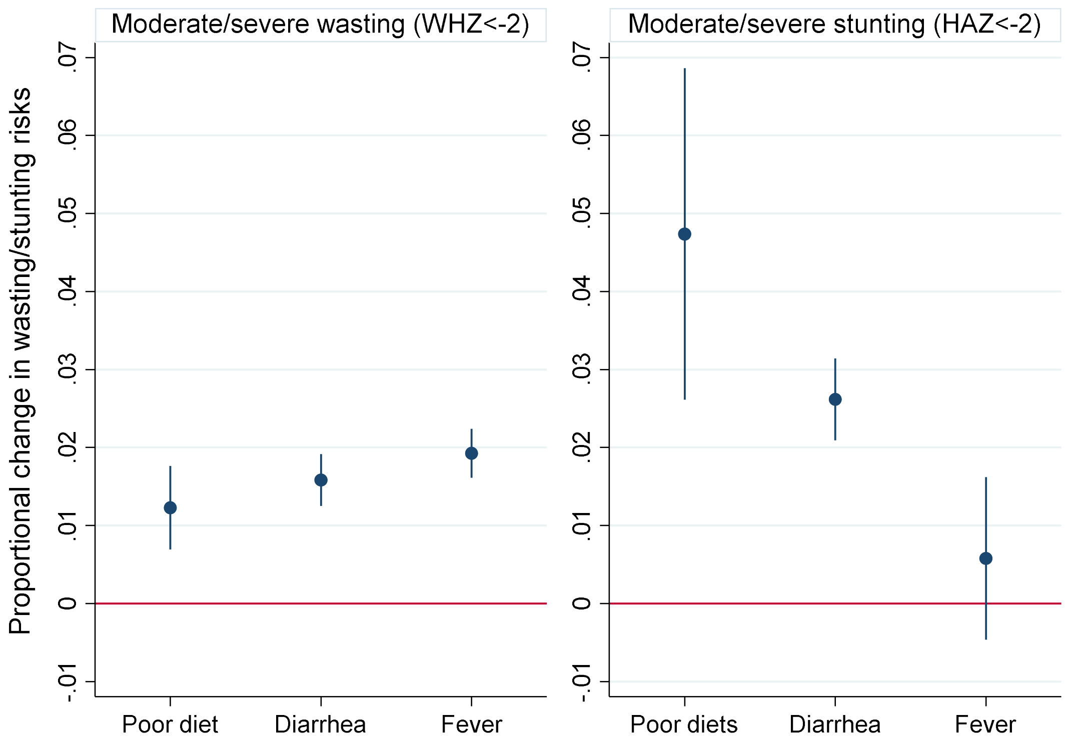

Notes: 95% confidence intervals based on standard errors clustered at the country level. Regressions are weighted (See Methods and Materials). The results show associations from two separate regressions for wasting and severe wasting. Coefficients represent the proportional change in wasting risks from a 5% increase in the real food price index over the past 3 months. The model also incorporates an extensive set of controls described in the Methods and Materials, including DHS variables, and various temporal effects and country fixed effects.

Supplementary Figure 5. Weighted multivariate linear probability coefficients of wasting and severe wasting as a function of both food inflation and total inflation over the past 3 months

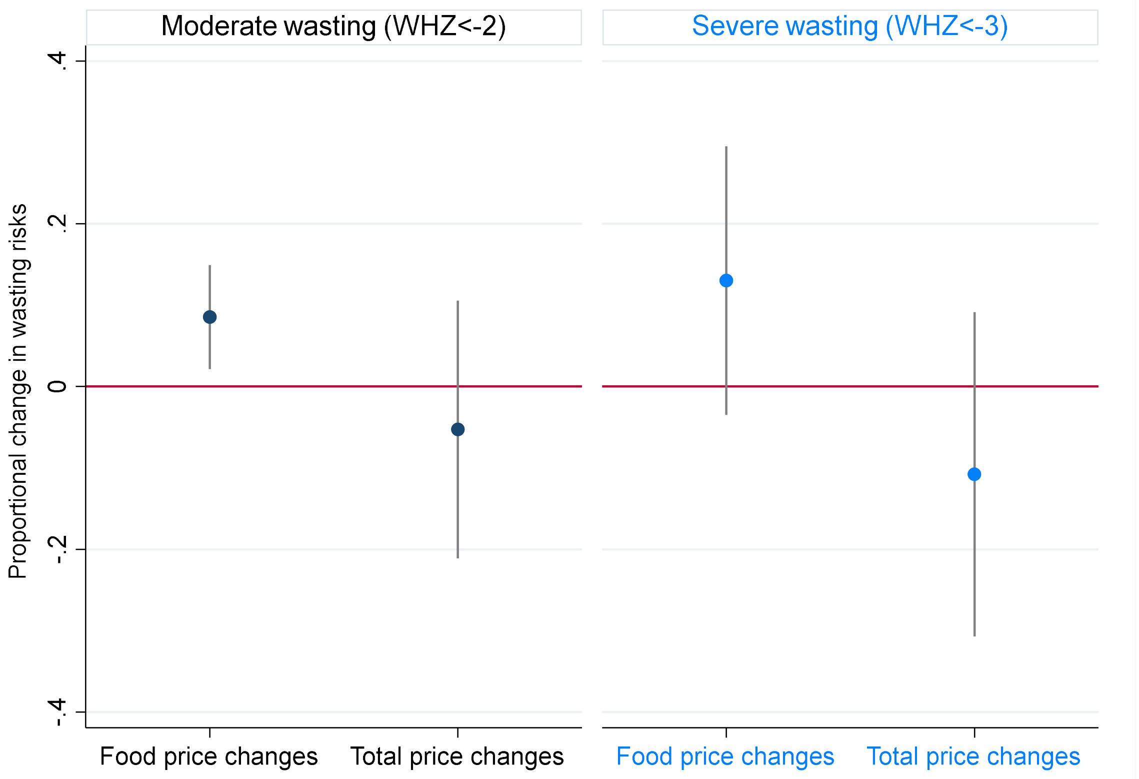

Notes: 95% confidence intervals based on standard errors clustered at the country level are reported in parentheses. Regressions are weighted (See Methods and Materials). Note that the results are not highly sensitive to inclusion/exclusion of either inflation measure. The model also incorporates an extensive set of controls described in the Methods and Materials, including DHS variables, and various temporal effects and country fixed effects.

Supplementary Figure 6. Weighted multivariate linear probability coefficients of stunting and severe stunting for children 24-59 months as a function of food inflation and total inflation at various stages of the first 1000 days of life

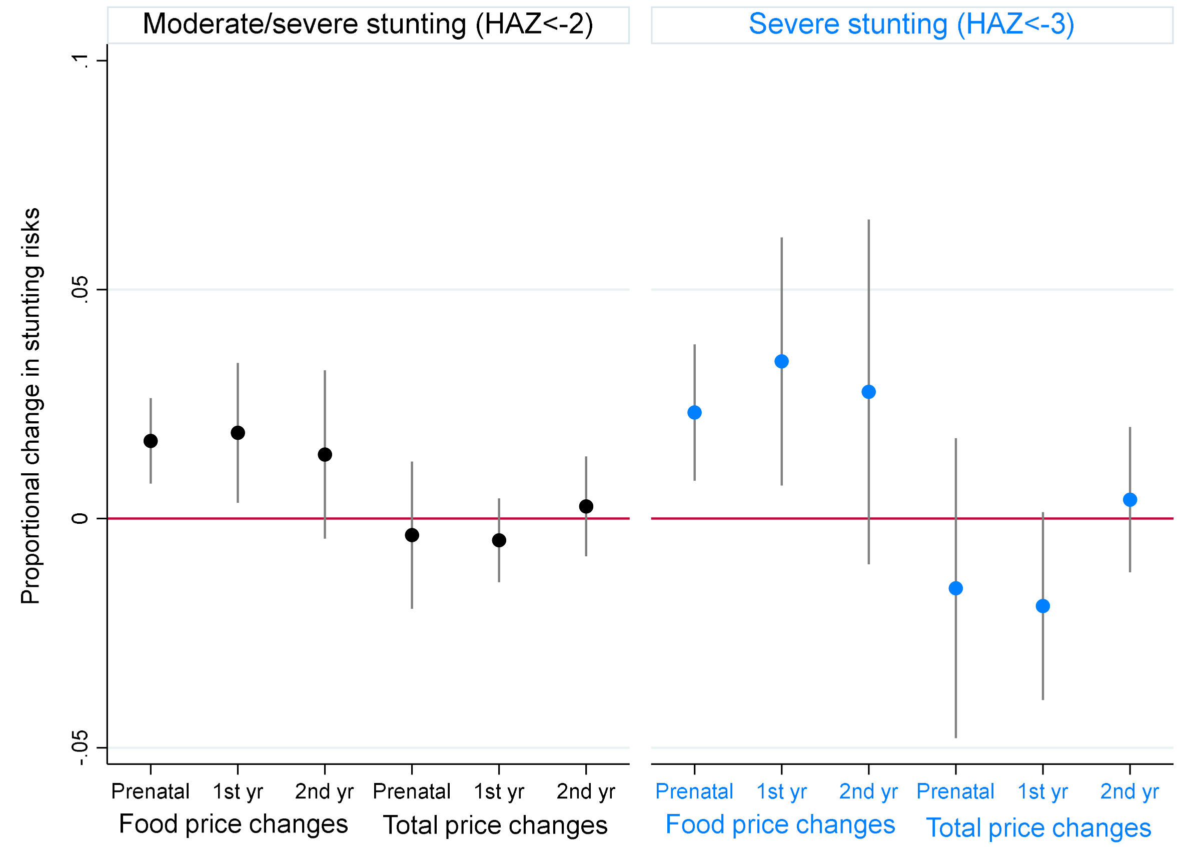

Notes: 95% confidence intervals based on standard errors clustered at the country level are reported in parentheses. Regressions are weighted (See Methods and Materials). However, the results are not sensitive to inclusion/exclusion of either inflation measure. The model also incorporates an extensive set of controls described in the Methods and Materials, including DHS variables, and various temporal effects and country fixed effects.
