# Supplementary material for: Food inflation and child undernutrition in low and middle income countries
Source: Nat Commun. 2023 Sep 16;14:5761. doi: 10.1038/s41467-023-41543-9 (PMC10505228; doi:10.1038/s41467-023-41543-9)
Supplement: Supplementary file 3 — Reporting Summary [file 41467_2023_41543_MOESM3_ESM.pdf]

nature portfolio

Corresponding author(s): Derek Headey

Last updated by author(s): Jul 29, 2023

Reporting Summary

Nature Portfolio wishes to improve the reproducibility of the work that we publish. This form provides structure for consistency and transparency in reporting. For further information on Nature Portfolio policies, see our Editorial Policies and the Editorial Policy Checklist.

Statistics

For all statistical analyses, confirm that the following items are present in the figure legend, table legend, main text, or Methods section.

n/a	Confirmed	
☐	☒	The exact sample size (n) for each experimental group/condition, given as a discrete number and unit of measurement
☒	☐	A statement on whether measurements were taken from distinct samples or whether the same sample was measured repeatedly
☐	☒	The statistical test(s) used AND whether they are one- or two-sided<br>*Only common tests should be described solely by name; describe more complex techniques in the Methods section.*
☐	☒	A description of all covariates tested
☐	☒	A description of any assumptions or corrections, such as tests of normality and adjustment for multiple comparisons
☐	☒	A full description of the statistical parameters including central tendency (e.g. means) or other basic estimates (e.g. regression coefficient) AND variation (e.g. standard deviation) or associated estimates of uncertainty (e.g. confidence intervals)
☐	☒	For null hypothesis testing, the test statistic (e.g. F, t, r) with confidence intervals, effect sizes, degrees of freedom and P value noted<br>*Give P values as exact values whenever suitable.*
☒	☐	For Bayesian analysis, information on the choice of priors and Markov chain Monte Carlo settings
☒	☐	For hierarchical and complex designs, identification of the appropriate level for tests and full reporting of outcomes
☐	☒	Estimates of effect sizes (e.g. Cohen's d, Pearson's r), indicating how they were calculated

Our web collection on statistics for biologists contains articles on many of the points above.

Software and code

Policy information about availability of computer code

Data collection	STATA Version 17 was used to merge together Demographic Health Survey data with FAO consumer food price data and other national level covariates.
Data analysis	STATA Version 17 was used to calculate descriptive statistics and implemented non-parametric and parametric regression analysis.

For manuscripts utilizing custom algorithms or software that are central to the research but not yet described in published literature, software must be made available to editors and reviewers. We strongly encourage code deposition in a community repository (e.g. GitHub). See the Nature Portfolio guidelines for submitting code & software for further information.

Data

Policy information about availability of data

All manuscripts must include a data availability statement. This statement should provide the following information, where applicable:

- Accession codes, unique identifiers, or web links for publicly available datasets
- A description of any restrictions on data availability
- For clinical datasets or third party data, please ensure that the statement adheres to our policy

This analysis uses three sources of data which were merged into a single dataset. The main dataset is a child level dataset compiling 130 national level Demographic Health Surveys from 44 countries implemented over 2000-2021 for children 0-59 months of age. These data were downloaded from https://dhsprogram.com/data/available-datasets.cfm and are freely available after registration on the DHS website. The individual survey rounds are listed in Appendix Table A1 in our paper. These 130 surveys were then stacked and variables from FAO were then merged to the year and month of interview or a child's birth month. FAO data is freely

accessible here: https://www.fao.org/faostat/en/#data/CP. Our data availability statement reads as follows: "The Demographic Health Survey data used in this analysis are available under restricted access as per the data access policies of the DHS Program, though access request after registering on the DHS website: https://dhsprogram.com/data/Using-Datasets-for-Analysis.cfm. The FAO consumer food price data are publicly available at https://www.fao.org/faostat/en/#data/CP. Source data are also provided with this paper. The analysis in this paper also conforms to the Guidelines for Accurate and Transparent Health Estimates Reporting: the GATHER statement.57"

Human research participants

Policy information about studies involving human research participants and Sex and Gender in Research.

Reporting on sex and gender	We use secondary datasets pooling together Demographic Health Surveys that collected anthropometric and other data for children 0-59 months of both sexes. We tested for differential impacts of food inflation on wasting for male and female children by adding interaction terms to our main regression model.
Population characteristics	Children 0-59 months of age in 130 national Demographic Health Surveys from 44 low and middle income countries.
Recruitment	We use secondary datasets. Recruitment and survey design are described on the Demographic Health Surveys website: https://dhsprogram.com/Methodology/index.cfm
Ethics oversight	We use secondary datasets so ethical oversight was not required but was undertaken by the DHS. The Methods section now clarifies this as follows: "The DHS Procedures and questionnaires for standard DHS surveys have been reviewed and approved by ICF Institutional Review Board (IRB), while country-specific DHS survey protocols are reviewed by the ICF IRB and typically by an IRB in the host country to ensure that the survey complies with the U.S. Department of Health and Human Services regulations for the protection of human subjects (45 CFR 46), while the host country IRB ensures that the survey complies with laws and norms of the nation."

Note that full information on the approval of the study protocol must also be provided in the manuscript.

Field-specific reporting

Please select the one below that is the best fit for your research. If you are not sure, read the appropriate sections before making your selection.

☐ Life sciences ☒ Behavioural & social sciences ☐ Ecological, evolutionary & environmental sciences

For a reference copy of the document with all sections, see nature.com/documents/nr-reporting-summary-flat.pdf

Behavioural & social sciences study design

All studies must disclose on these points even when the disclosure is negative.

Study description	This is a quantitative study linking together secondary datasets - micro data from household surveys with macroeconomic indicators from national accounts databases - to test whether national income growth rates affect changes in wasting risks (low weight-for-height) among children 0-59 months of age, as well as child stunting risks for children 24-59 months of age.
Research sample	We use a sample of children 0-59 months of age surveyed in 130 nationally representative Demographic Health Surveys (DHS) implemented in 44 low and middle income countries between 2000-2021, and described here: https://dhsprogram.com/. The sample is representative of the 44 countries in question but not of all low and middle income countries. The sample is chosen based on the availability of the DHS. Our primary outcome is a child's wasting status, defined as whether their weight-for-height z scores (relative to international reference standards) are significantly below various thresholds (WHZ<-2, <-3). Wasting status is then linked to real food price changes in the 3 months prior to each child's recorded measurement year and month. We also conduct an analysis of child stunting, linking inflation in utero and the first two years after birth to stunting status when the child 24-59 months of age.
Sampling strategy	Demographic Health Surveys collection nationally representative samples through a complex two-stage stratified design, with household weights used to derive nationally representative sampling statistics. The households in a survey area are stratified according to type of residence (urban-rural) crossed by administrative/geographical regions. In the first stage, a number of primary sampling units (PSUs), or clusters, are selected from a sampling frame independently in each stratum. The sampling frame is usually a complete list of enumeration areas (EAs) created in a recent population census. After the selection of EAs and before the second-stage selection, a household listing and mapping operation is conducted in each of the selected EAs. This operation updates the outdated population information in the sampling frame and provides a list of all of the households residing in each EA with a location map. In the second stage, a fixed number of households are selected from the newly constructed household list in each of the selected EAs, and all household members in a certain age group (e.g., all women age 15-49 and all men age 15-59) in the selected household are selected for the survey. Further details are available at https://dhsprogram.com/pubs/pdf/WP30/WP30.pdf
Data collection	We used secondary datasets collected by the DHS program. The researchers were not blinded to the study hypothesis.
Timing	Demographic Health Surveys with the full range of anthropometric data for children 0-59 months of age were first collected in 2000 and most recently collected in 2021. The timing of surveys in each country differs, and is reported for each of the 130 surveys in Supplement Table 3.

Data exclusions	Demographic Health Surveys that did not collect anthropometric data for all children 0-59 months were excluded, along with surveys conducted before 2000 as the FAO price indices are only available from January 2000 onwards.
Non-participation	Non-participation is not directly relevant to this study as it uses secondary datasets. Non-participation (non-response rates) are reported in the original DHS survey reports for each of the 130 surveys. Non-response rates in the DHS are generally less than 10%, as noted in the following report: https://dhsprogram.com/pubs/pdf/WP30/WP30.pdf
Randomization	Randomization is not applicable to this study. The analysis was observational rather than experimental, so randomization was not applicable to the data analysis. Randomization was also not used in the DHS data collection as DHS surveys are two-stage surveys in which the first stage is a systematic sampling with probability proportional to the EA size and the second stage is a systematic sampling of equal probability and fixed size across the enumeration areas. This is described in the following report: https://dhsprogram.com/pubs/pdf/WP30/WP30.pdf

Reporting for specific materials, systems and methods

We require information from authors about some types of materials, experimental systems and methods used in many studies. Here, indicate whether each material, system or method listed is relevant to your study. If you are not sure if a list item applies to your research, read the appropriate section before selecting a response.

Materials & experimental systems

n/a	Involved in the study
☒	☐ Antibodies
☒	☐ Eukaryotic cell lines
☒	☐ Palaeontology and archaeology
☒	☐ Animals and other organisms
☒	☐ Clinical data
☒	☐ Dual use research of concern

Methods

n/a	Involved in the study
☒	☐ ChIP-seq
☒	☐ Flow cytometry
☒	☐ MRI-based neuroimaging
